# Supplementary material for: Preadaptation of pandemic GII.4 noroviruses in unsampled virus reservoirs years before emergence
Source: Virus Evol. 2020 Nov 21;6(2):veaa067. doi: 10.1093/ve/veaa067 (PMC7751145; doi:10.1093/ve/veaa067)
Supplement: veaa067_Supplementary_Data [file veaa067_supplementary_data.zip › suppl_data/Ruis.supplemental.1.final.docx]

**Supplementary Text and Figures**

Supplementary Text

*Intergenic recombination*

Recombination between noroviruses frequently occurs close to open reading frame boundaries , including within GII.4 (Eden et al. 2013). We observe extensive topological differences between the RdRp, VP1 and VP2 trees in well supported regions, concurring with this previous evidence. In addition to the previously reported acquisition of a Den Haag 2006-like VP2 by the Osaka 2007 VP1 (Eden et al. 2013), we also find that that Apeldoorn lineage acquired a Yerseke 2006-like VP2 in 2003, prior to diverging into the Apeldoorn 2007, New Orleans 2009 and Sydney 2012 variants (Figure S10, Table S7). Multiple recombination events have occurred between the RdRp and VP1. Consistent with previous results (Eden et al. 2013), we infer that Asia 2003 acquired a GII.P12 RdRp and Osaka 2007 acquired a GII.P31 RdRp (Table S7). Multiple recombination events are required to explain the acquisition of RdRp and VP2 regions by the Apeldoorn lineage VP1 (Figure S10). A common ancestor of the New Orleans 2009 and Sydney 2012 (and possibly the Apeldoorn 2007) variants acquired a Yerseke 2006-like RdRp in 2004 (Figure S10, Table S7). While it was not possible to conclusively resolve the recombination events within the Apeldoorn lineage, Figure S10 depicts two plausible scenarios. As previously reported, the Sydney 2012 variant circulated commonly with both the GII.P4 New Orleans 2009-like RdRp and the GII.P31 RdRp (Wong et al. 2013). At least three independent recombination events are required to explain the distribution of sequences with the GII.P31 RdRp in the Sydney 2012 VP1 tree (Figure S7). Importantly, each of the recombination events where the two contributing variants could be identified occurred years prior to the pandemic or epidemic emergence of the contributing variants (Table S7).

**References**

Eden J-S, Tanaka MM, Boni MF, Rawlinson WD, White PA. 2013. Recombination within the pandemic norovirus GII.4 lineage. J. Virol. 87:6270–6282.

Lindesmith LC, Beltramello M, Donaldson EF, Corti D, Swanstrom J, Debbink K, Lanzavecchia A, Baric RS. 2012. Immunogenetic mechanisms driving norovirus GII.4 antigenic variation. PLoS Pathog. 8.

Tohma K, Lepore CJ, Gao Y, Ford-Siltz LA, Parra GI. 2019. Population Genomics of GII.4 Noroviruses Reveal Complex Diversification and New Antigenic Sites Involved in the Emergence of Pandemic Strains. MBio 10.

Wong THN, Dearlove BL, Hedge J, Giess AP, Piazza P, Trebes A, Paul J, Smit E, Smith EG, Sutton JK, et al. 2013. Whole genome sequencing and de novo assembly identifies Sydney-like variant noroviruses and recombinants during the winter 2012/2013 outbreak in England. Virol. J. 10:335.

Fig. S1.

Temporal MCC trees of the RdRp and VP2. The temporal history of the RdRp and VP2 was reconstructed using BEAST. As with the VP1 tree in Figure 1, the trees of these genomic regions exhibit a high degree of unsampled diversity with a large number of long branches. Each variant diverged from all other sampled variants years prior to pandemic/epidemic emergence. Variants are labelled in different colors. Posterior supports are shown on trunk nodes.

**Fig S2.**

**Comparison of the early part of the GII.4 phylogenetic tree between this study and the study of Bok et al (Bok et al. 2009). We include an additional GII.4 sequence collected in 1978 that diverges from a more ancestral node (Node 1) in the GII.4 phylogenetic tree than any sequence included in Bok et al’s analysis. Our analysis therefore finds an earlier common ancestor date for the GII.4 genotype than Bok et al. We obtain a highly similar date for the phylogenetic node (Node 2) that was the common ancestor of the GII.4 genotype in Bok et al’s analysis.**

**Fig S3**

(**A**) Identification of pre-pandemic and pre-epidemic sequences. We identified all available GII.4 norovirus sequences with a reported collection date earlier than the start of the year of pandemic/epidemic emergence of the genotyped variant. We verified the collection date of each sequence using Bayesian tip dating (see methods) and identified 31 pre-pandemic/pre-epidemic sequences from the Farmington Hills 2002, Hunter 2004, Osaka 2007, New Orleans 2009 and Sydney 2012 variants. Each sequence is represented here by a diamond at the date at which the sequence was collected, with the color of the diamond representing the continent on which the sequence was collected. The shaded area represents the period of pandemic (purple) or epidemic (blue) circulation. (**B**) The age of the infected patient was reported for 16 of the 31 pre-pandemic/pre-epidemic sequences. 15 of these 16 sequences were collected from children. (**C**) Putative pre-pandemic/pre-epidemic sequences exhibit a level of divergence consistent with their reported collection date. We reconstructed a nucleotide maximum likelihood tree on all available GII.4 VP1 sequences and rooted to maximize the correlation between root-to-tip distance and collection date. Each panel contains all of the sequences from the corresponding variant. Putative pre-pandemic/pre-epidemic sequences are shown in magenta, the remaining sequences are shown in grey. The black dashed line is a regression line between root-to-tip distance and collection date calculated on all GII.4 VP1 sequences. The red vertical line represents the start of the year in which the variant emerged as a pandemic or epidemic.

**Fig. S4**

Evolutionary dynamics of New Orleans 2009 and Sydney 2012. Summary of phylodynamic analyses of New Orleans 2009 (A and C) and Sydney 2012 (B and D). (A and B) Temporal MCC trees of all available New Orleans 2009 (n=466) and Sydney 2012 (n=533) P2 domain sequences reconstructed using BEAST. (C and D) Bayesian skyline plots showing a measure of relative genetic diversity through time. The solid black line is the median value and the grey shaded area the 95% HPD. The vertical red and blue lines represent the time of onset of the New Orleans 2009 and Sydney 2012 pandemics, as estimated from the Bayesian skyline plots. By this time each variant had already diverged into a large number of lineages.

**Fig. S5.**

Sydney 2012 could resist anti-New Orleans 2009 murine mAbs by 2003. We tested the ability of mouse mAbs raised against New Orleans 2009 to block interaction of $\mathrm{Sydney}_{\mathrm{All}}^{\mathrm{Anc}},$ $\mathrm{Sydney}_{\mathrm{Pand}}^{\mathrm{Anc}},$ New Orleans^Ref^ and Sydney^Ref^ VLPs with pig gastric mucin (PGM). The ancestral Sydney 2012 VLPs resisted mAbs raised against blockade epitopes of New Orleans^Ref^ to a comparable or greater degree compared with the Sydney^Ref^ VLP. Markers represent the mean and error bars the 95% confidence intervals. * significantly different from New Orleans^Ref^, # significantly different from Sydney^Ref^ (Dunnett multiple comparison test).
