# Supplementary material for: Preadaptation of pandemic GII.4 noroviruses in unsampled virus reservoirs years before emergence
Source: Virus Evol. 2020 Nov 21;6(2):veaa067. doi: 10.1093/ve/veaa067 (PMC7751145; doi:10.1093/ve/veaa067)
Supplement: veaa067_Supplementary_Data [file veaa067_supplementary_data.zip › suppl_data/Ruis.supplemental.2.final.docx]

**Supplementary Figures**

**Fig. S6.**

Substitutions potentially important for the pre-adaptation of Sydney 2012. (A and C) We identified the distribution of amino acid residues within New Orleans 2009 and Sydney 2012 at each site in VP1 (A) and VP2 (C) that underwent a substitution leading to Sydney 2012. The residues at each site are shown between the respective pie charts. In VP1, only sites 310, 368 and 373 exhibit different amino acid residues in Sydney 2012 compared with New Orleans 2009. (B and D) Conservation of sites across the Sydney 2012 clade. Each tip within maximum likelihood trees of all available New Orleans 2009 and Sydney 2012 VP1 sequences is colored by the amino acid residue within that sequence at the respective site. Each of the sites is conserved downstream of $\mathrm{Sydney}_{\mathrm{Pand}}^{\mathrm{Anc}},$, with the exception of VP1 site 373 which changes between histidine and arginine, likely on multiple occasions. Black tips indicate that the site was not sequenced within that sample.

**Fig. S7.**

(A) The tips in the GII.4 Sydney 2012 VP1 tree are coloured by the associated nonstructural polyprotein genotype. This variant co-circulated with the GII.P31 and GII.P4 nonstructural polyproteins throughout its pandemic period and has more recently circulated with the GII.P16 nonstructural polyprotein. (B) The GII.4 Sydney 2012 clade in the VP1 tree in Figure 1 is shown. Tip branches are colored by the RdRp found with that sequence: red – GII.P4 New Orleans 2009-like, blue – GII.P31. Acquisition of the GII.P31 RdRp was inferred to have occurred along the three branches marked with asterisks. (C) The GII.4 Sydney[GII.P31] clade in the RdRp tree in Figure S1 is shown. While the sequences marked with orange and blue rectangles are monophyletic in the VP1 tree, these sequences cluster apart in the RdRp tree and were therefore acquired in separate recombination events. The RdRp marked with a blue rectangle clusters within the GII.4 Sydney[GII.P31] clade, strongly suggesting that this RdRp was acquired from a virus with a Sydney 2012 VP1, indicating cocirculation of Sydney 2012 viruses with the GII.P4 New Orleans 2009-like RdRp and Sydney 2012 viruses with the GII.P31 RdRp. Posterior supports are shown at key nodes

**Fig. S8.**

Temporal evolutionary signal within the RdRp, VP1 and VP2. We reconstructed a nucleotide maximum likelihood tree for each genomic region. Plotted here is the correlation between root-to-tip distance and collection date. The R2 correlation is shown, statistical significance of this correlation was calculated using non-parametric bootstrapping where the collection dates were randomly resampled and the R2 correlation re-calculated 1000 times. Points in the RdRp plot are coloured by RdRp genotype.


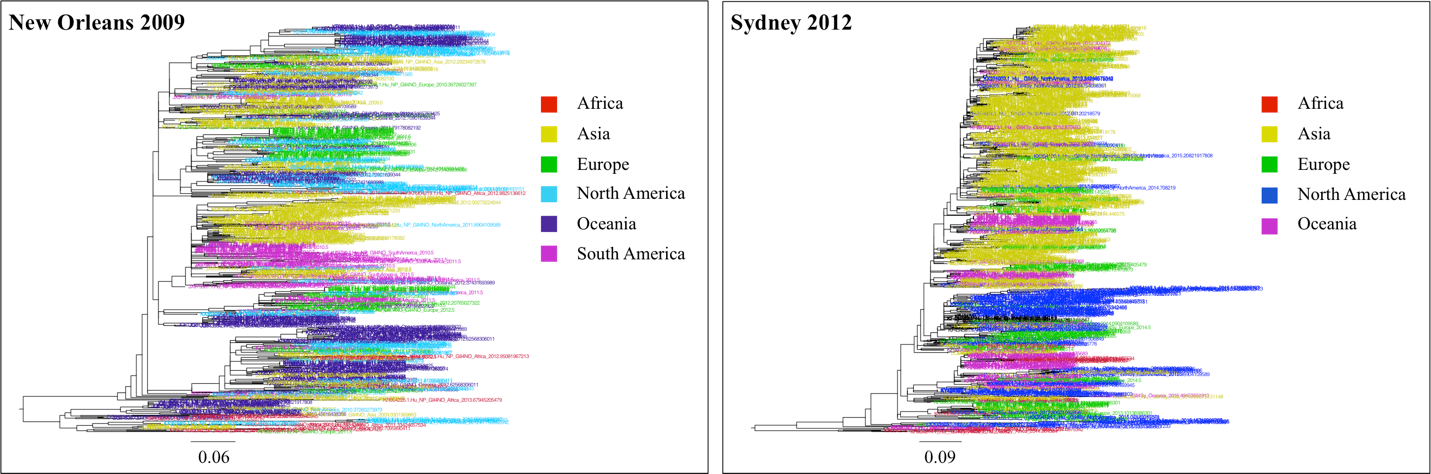


**Fig. S9.**

Interspersion of sequences from each continent within the New Orleans 2009 and Sydney 2012 phylogenetic trees. We reconstructed a nucleotide maximum likelihood tree for the complete New Orleans 2009 and Sydney 2012 VP1 datasets. Each tip label is colored by the continent on which the sequence was collected. The interspersion of sequences from each continent throughout the tree led us to down-sample the sequences from over-represented continents. The scale bar shows the expected number of nucleotide substitutions per site.

**Fig. S10.**

Plausible scenarios for recombination within the Apeldoorn lineage. Shown are two hypothetical scenarios for the acquisition of RdRp and VP2 genomic regions by the Apeldoorn lineage VP1 (consisting of Apeldoorn 2007, New Orleans 2009 and Sydney 2012) that are consistent with the tree topologies and divergence dates in Figures 1 and S1. The tree backbones are from the VP1 tree in Figure 1 in which the relationship between Apeldoorn 2007, New Orleans 2009 and Sydney 2012 is uncertain. Therefore each scenario depicts a hypothetical relationship between these variants that fits the branching patterns and inferred ancestor dates within the RdRp, VP1 and VP2 phylogenetic trees. Arrows represent a recombination event in which a RdRp or VP2 genomic region is obtained, with the arrow pointing from donor variant to recipient variant. Triangles at the end of branches represent the remaining lineages within the variant. (A) This scenario involves the acquisition of a Yerseke 2006-like VP2 prior to the divergence of the variants within the lineage. The relationship between Apeldoorn 2007, New Orleans 2009 and Sydney 2012 is the same as the well-supported relationship within the VP2 tree, with Sydney 2012 diverging first. The Yerseke 2006 RdRp is acquired prior to divergence of variants within the Apeldoorn lineage and Apeldoorn 2007 acquires a Hunter 2004-like RdRp after its divergence from New Orleans 2009. (B) This scenario involves acquisition of the Yerseke 2006 VP2 and a Hunter 2004-like RdRp prior to divergence of the variants within the Apeldoorn lineage. Here, Apeldoorn 2007 branches first and the Yerseke 2006-like RdRp is acquired leading to the common ancestor of New Orleans 2009 and Sydney 2012. Sydney 2012 acquires its VP2 region from an early Apeldoorn lineage virus that has persisted, explaining the divergence of Sydney 2012 in the VP2 tree. In both scenarios, Sydney 2012 acquires the GII.P31 RdRp after divergence from Apeldoorn 2007 and New Orleans 2009. Scenario B requires one more recombination event than scenario A and requires the persistence of an unsampled early Apeldoorn lineage.
