# Supplementary material for: Preadaptation of pandemic GII.4 noroviruses in unsampled virus reservoirs years before emergence
Source: Virus Evol. 2020 Nov 21;6(2):veaa067. doi: 10.1093/ve/veaa067 (PMC7751145; doi:10.1093/ve/veaa067)
Supplement: veaa067_Supplementary_Data [file veaa067_supplementary_data.zip › suppl_data/Ruis.supplemental.3.final.docx]

**Supplementary Tables**

| **Genome region** | **Log10 Bayes factor rejecting strict clock** | **Evolutionary rate, x10^-3^ substitutions/site/year (95% HPD)** | **Root date (95% HPD)** | **GII.P4 RdRp ancestor date (95% HPD)** |
| --- | --- | --- | --- | --- |
| RdRp | 54.89 | 6.67 (5.84, 7.53) | 1941 (1922, 1958) | 1979 (1975-1982) |
| VP1 | 122.97 | 6.83 (6.03, 7.68) | 1943 (1919, 1965) | N/A |
| VP2 | 44.23 | 6.11 (5.35, 6.93) | 1949 (1934, 1963) | N/A |

**Table S1.**

Summary of Bayesian MCMC results. Mean values and 95% HPD intervals were calculated by combining the complete posterior distribution for the parameter of interest from each of the subsampled datasets. The Log10 Bayes factor is the support for rejecting the strict clock model (single substitution rate across the tree) in favor of the relaxed lognormal clock model (each branch on the phylogenetic tree can have a different substitution rate). This Bayes factor therefore suggests variation in the substitution rate along different branches within each dataset.

| **GII.4 Variant** | **US95/96** | **Farmington Hills 2002** | **Asia 2003** | **Hunter 2004** | **Yerseke 2006** | **Den Haag 2006** | **Osaka 2007** | **Apeldoorn 2007** | **New Orleans 2009** | **Sydney 2012** |
| --- | --- | --- | --- | --- | --- | --- | --- | --- | --- | --- |
| **US95/96** |  | 1992 (1989, 1994) | 1992 (1989, 1994) | 1992 (1989, 1994) | 1992 (1989, 1994) | 1992 (1989, 1994) | 1992 (1989, 1994) | 1992 (1989, 1994) | 1992 (1989, 1994) | 1992 (1989, 1994) |
| **Farmington Hills 2002** |  |  | 1997 (1995, 1999) | 1996 (1994, 1998) | 1996 (1994, 1998) | 1997 (1994, 1999) | 1994 (1991, 1996) | 1996 (1994, 1998) | 1996 (1994, 1998) | 1996 (1994, 1998) |
| Asia 2003 |  |  |  | 1996 (1994, 1998) | 1996 (1994, 1998) | 1996 (1994, 1998) | 1994 (1991, 1996) | 1996 (1994, 1998) | 1996 (1994, 1998) | 1996 (1994, 1998) |
| **Hunter 2004** |  |  |  |  | 2001 (1999, 2002) | 1997 (1995, 1999) | 1994 (1991, 1996) | 1999 (1996, 2001) | 1999 (1996, 2001) | 1999 (1996, 2001) |
| Yerseke 2006 |  |  |  |  |  | 1997 (1995, 1999) | 1994 (1991, 1996) | 1999 (1996, 2001) | 1999 (1996, 2001) | 1999 (1996, 2001) |
| **Den Haag 2006** |  |  |  |  |  |  | 1994 (1991, 1996) | 1997 (1995, 2000) | 1997 (1995, 2000) | 1997 (1995, 2000) |
| Osaka 2007 |  |  |  |  |  |  |  | 1994 (1991, 1996) | 1994 (1991, 1996) | 1994 (1991, 1996) |
| Apeldoorn 2007 |  |  |  |  |  |  |  |  | 2004 (2002, 2005) | 2003 (2002, 2005) |
| **New Orleans 2009** |  |  |  |  |  |  |  |  |  | 2004 (2002, 2005) |

**Table S2.**

Summary of variant divergence times. We calculated the date at which each pair of GII.4 variants diverged in each tree in the VP1 posterior distribution and calculated the mean and 95% HPD of this distribution. Each variant diverges from all of the other sampled variants years prior to pandemic or epidemic emergence, indicating variants evolved independently for years prior to emergence.

**Table S3.**

Summary of pre-pandemic and pre-epidemic GII.4 sequences. Summary of the 31 sequences with a reported collection date earlier than the year of pandemic/epidemic emergence where the reported collection date was supported by tip dating (see methods). These sequences were taken to be true pre-pandemic/pre-epidemic sequences. While the estimated collection date is here reported to the year, we compared the reported and estimated collection dates to the most precise date possible. Therefore where the reported collection date was given to the nearest day or month, the 95% HPD of the estimated collection date overlaps with that day or month. The proportion of the complete VP1 sequence present in each putative pre-pandemic/pre-epidemic sequence is shown, AA – amino acid.

**Table S4.**

Summary of the 19 sequences with a reported collection date earlier than the year of pandemic/epidemic emergence where the reported collection date was not supported by tip dating (see methods). These sequences were not taken to be true pre-pandemic/pre-epidemic sequences. Possible reasons for this discrepancy include mis-reporting of the collection date, sample contamination, sample mis-labelling and inaccurate estimation of the collection date. The 95% HPD of the estimated collection date of sequence EU916958.1 does not overlap with the reported collection date to the level of the day. AA – amino acid.

**Table S5.**

The common ancestor date of each GII.4 variant is shown for the RdRp, VP1 and VP2. This was estimated by combining the posterior distribution of the common ancestor date for each subsampled dataset. The common ancestor of the Sydney 2012 RdRp is the common ancestor of the GII.P31 RdRps found with the Sydney 2012 VP1.

| **Variant** | **Nonstructural polyprotein** | **VP1** | **VP2** |
| --- | --- | --- | --- |
| Farmington Hills 2002 | P44S, I52T, M246V, R278K, V525I, T650A, K729R, A782T, N787S, K807R, A850V, S1270N, K1552R | *S9N*, D298N, K329R, S355D, V365I, T368N, S394G, N407S, A534T | N23S, K80E, Q83R, A97S, S155F, T158V |
| Hunter 2004 | Y759H, S760N, *K807R* | Node 1 - H297Q, N372S, K382R, N412D, T425S  Node 2 - N9T, N407D, G413S, S425T, A465S | I101V, L140S, S162A |
| Den Haag 2006 | G27K, V28M, L29F, I79V, V85A, A104T, A261T, L283I, Y327F, A336V, V525I, T650A, R738K, A782T, T791A, V830I, M833V, D983E, S1182G, R1420K, N1575D, T1618S, A1642T | T15A, P174S, H297R, Q306L, R339K, S352Y, V356A, H357P, N372E, G378H, N407S, G413V | V33I, E34D, T144I, A148T, T149P, V150T, P159S, V168I, S187N, K262R |
| New Orleans 2009 | *V525I*, I750V, *V779I*, K1210T, *S1610P*, E1613G | T294A, A340T, A359S | *H130R*, T164I |
| Sydney 2012 | Circulated with multiple nonstructural polyprotein genotypes | $\mathrm{Sydney}_{\mathrm{All}}^{\mathrm{Anc}}$ – T15A  $\mathrm{Sydney}_{\mathrm{Pand}}^{\mathrm{Anc}}$- *H297R*, S310N, A368E, N373H, *N393S*, *A395T*, *A539V*, *L540V* | $\mathrm{Sydney}_{\mathrm{All}}^{\mathrm{Anc}}$- A148D  $\mathrm{Sydney}_{\mathrm{Pand}}^{\mathrm{Anc}}$- T158K, N205S |

**Table S6.**

Summary of nonsynonymous substitutions leading to the five most recent pandemic GII.4 variants. We used ancestral reconstruction to identify the nonsynonymous substitutions that occurred along the phylogenetic branch leading to the common ancestor of each of the five most recent pandemic GII.4 variants within each genomic region. These substitutions delimit that variant from the other, typically unsampled, lineages present at the time of pandemic emergence and so are likely to encode the characteristic(s) that enabled pandemic emergence. Substitutions in italics are unlikely to have been important for pandemic emergence, as the residue(s) at these sites was the same within the preceding pandemic variant. Red substitutions occurred at VP1 sites within known blockade epitopes (Lindesmith et al. 2012, Tohma et al. 2019). There are two potential common ancestor nodes for Hunter 2004; the substitutions leading to each of these nodes are shown here.

| **VP1 variant** | **RdRp or VP2 acquired** | **Inferred date of recombination event (95% HPD)** |
| --- | --- | --- |
| Asia 2003 | GII.P12 RdRp | March 1999 (March 1996-October 2001) |
| Osaka 2007 | GII.P31 RdRp | June 2000 (October 1994-January 2006) |
| Osaka 2007 | Den Haag 2006-like VP2 | March 2005 (December 2003-August 2006) |
| Apeldoorn lineage | Yerseke 2006-like VP2 | March 2004 (February 2003-April 2005) |
| Apeldoorn lineage | Yerseke 2006-like RdRp | July 2004 (January 2003-November 2005) |
| Sydney 2012 | GII.P31 RdRp | May 2010 (July 2009-February 2011)  January 2012 (July 2011-May 2012)  February 2012 (August 2011-July 2012) |

**Table S7.**

Summary of recombination events and dates in the GII.4 lineage. Shown are the dates at which the GII.4 VP1 variants acquired a new RdRp or VP2. Recombination events were initially identified based on well-supported topological differences between the VP1 tree and the RdRp or VP2 tree. The date of the recombination event was calculated from the posterior distribution of trees from the corresponding dataset by identifying the branch along which the recombination event occurred in each tree. Sydney 2012 acquired the GII.P31 RdRp in at least three independent recombination events. Additional recombination events likely occurred in the Apeldoorn lineage (which consists of the Apeldoorn 2007, New Orleans 2009 and Sydney 2012 variants). Further information on recombination within the Apeldoorn lineage is shown in Figures S6 and S9.

| **Most likely recombination breakpoint** | **GII.4 variant 5’ to breakpoint** | **GII.4 variant 3’ to breakpoint** | **Number of sequences** | **Accession numbers** |
| --- | --- | --- | --- | --- |
| 537 | Den Haag 2006 | New Orleans 2009 | 12 | KF712501.1, KF429790.1, KF712491.1, KF429762.1, KF712498.1, KF712505.1, KF712495.1, KF429785.1, KF429776.1, JX459900.1, KF712502.1, AB933738.1 |
| 537 | Den Haag 2006 | Apeldoorn 2007 | 1 | AB541362.1 |
| 537 | New Orleans 2009 | Apeldoorn 2007 | 1 | JX448566.1 |
| 314 | Den Haag 2006 | Apeldoorn 2007 | 1 | KF712492.1 |
| 314 | New Orleans 2009 | Den Haag 2006 | 3 | KF196287.1, AB933682.1, AB447434.1 |
| 314 | Unclear | Osaka 2007 | 1 | GQ845368.2 |
|  |  |  |  |  |

**Table S8.** Summary of putative recombination events within the GII.4 VP1. We identified putative recombination events using SBP. The most likely recombination breakpoint was defined as the position with the greatest gain in Akaike information criteria (AIC) when splitting the alignment on that alignment site compared with the null model. This breakpoint is therefore approximate. The number of sequences that cluster differently on either side of the putative breakpoint but cluster together on both sides of the putative breakpoint is shown with the variant within which the sequences cluster. Removal of the sequences in this table resulted in loss of the recombination signal.

| **Variant name** | **Number of RdRp sequences** | **Number of VP1 and VP2 sequences** | **Subsampled** |
| --- | --- | --- | --- |
| GII.4 1970s (GII.P1 RdRp) | 6 | 6 | No |
| Bristol 1993 | 2 | 2 | No |
| Camberwell 1994 | 5 | 5 | No |
| US95/96 | 3 | 3 | No |
| Lanzhou 2001 | 1 | 1 | No |
| Farmington Hills 2002 | 18 | 18 | No |
| Asia 2003 (GII.P12 RdRp) | 15 | 15 | No |
| Hunter 2004 | 17 | 17 | No |
| Yerseke 2006 | 12 | 12 | No |
| Den Haag 2006 | 551 | 559 | Yes, 41 sequences |
| Osaka 2007 (GII.P31 RdRp) | 5 | 5 | No |
| Apeldoorn 2007 | 37 | 31 | No |
| New Orleans 2009 | 141 | 134 | Yes, 41 sequences |
| Sydney 2012 (GII.P31 RdRp) | 36 | 41 | No |
| GII.4 could not assign strain | 3 | 3 | No |

**Table S9.**

Summary of the GII.4 sequences included in the dataset used to reconstruct the temporal history of the complete GII.4 genotype. The number of sequences from each GII.4 variant is shown following the removal of potentially recombinant sequences. The RdRp genotype is shown in parentheses for each GII.4 variant that is not found with the GII.P4 RdRp. Den Haag 2006 and New Orleans 2009 were randomly subsampled to the number of sequences present in the third most prevalent variant. Sydney 2012 and Osaka 2007 are found with the GII.P31 RdRp, therefore there are 41 GII.P31 sequences in the RdRp dataset.

**Table S10.**

Comparison of variant ancestor dates in each subsampled dataset. Samples 1, 2 and 3 contain a different random subsample of 41 sequences from Den Haag 2006 and 41 sequences from New Orleans 2009. The Sydney 2012 RdRp ancestor date is the common ancestor of the GII.P31 RdRps found with the GII.4 Sydney 2012 VP1.

**Table S11.**

Summary of the collection countries and continents of sequences used in phylogeographic analysis. The number of sequences from each country and from each continent is shown for the New Orleans 2009 and Sydney 2012 VP1 datasets. Sequences from Asia and Oceania were randomly subsampled in the New Orleans 2009 dataset and sequences from Asia were randomly subsampled in the Sydney 2012 dataset.

**Table S12.**

Summary of the datasets used in reconstruction of the Sydney 2012 ancestral VP1 sequences and to identify the nonsynonymous substitutions that occurred in VP1 and VP2 leading to each pandemic GII.4 variant. Strain typing was carried out using the norovirus genotyping tool. All variant assignments were confirmed by subsequent phylogenetic analyses. Variant names used are those returned by the norovirus genotyping tool for VP1.

**Table S13.**

Summary of the dataset used in reconstruction of the nonsynonymous substitutions that occurred leading to each pandemic GII.4 variant within the nonstructural polyprotein. Strain typing was carried out using the norovirus genotyping tool. All variant assignments were confirmed by subsequent phylogenetic analyses. Variant names are those returned by the norovirus genotyping tool.
